# Physical activity modulates mononuclear phagocytes in mammary tissue and inhibits tumor growth in mice

**DOI:** 10.7717/peerj.10725

**Published:** 2021-01-19

**Authors:** Donald M. Lamkin, Karen P. Bradshaw, Janice Chang, Ma’ayan Epstein, Jack Gomberg, Krupa P. Prajapati, Veronica H. Soliman, Thezia Sylviana, Yinnie Wong, Kouki Morizono, Erica K. Sloan, Steve W. Cole

**Affiliations:** 1 Norman Cousins Center for PNI, Semel Institute for Neuroscience, University of California, Los Angeles, CA, United States of America; 2 Department of Psychiatry & Biobehavioral Sciences, David Geffen School of Medicine, University of California, Los Angeles, CA, United States of America; 3 Jonsson Comprehensive Cancer Center, University of California, Los Angeles, CA, United States of America; 4 Department of Neuroscience, Stanford University School of Medicine, Stanford, CA, United States of America; 5 Divison of Hematology-Oncology, Department of Medicine, David Geffen School of Medicine, University of California, Los Angeles, CA, United States of America; 6 UCLA AIDS Institute, David Geffen School of Medicine, University of California, Los Angeles, CA, United States of America; 7 Monash Institute of Pharmaceutical Sciences, Monash University, Parkville, Victoria, Australia; 8 Division of Cancer Surgery, Peter MacCallum Cancer Centre-Victorian Comprehensive Cancer Centre, Melbourne, Victoria, Austalia

**Keywords:** Exercise oncology, Voluntary wheel running, Breast cancer, Innate immunity, Myeloid cells, Tumor immunology, Monocytes, Macrophages, Transcriptomics

## Abstract

The risk for breast cancer is significantly reduced in persons who engage in greater amounts of physical activity, and greater physical activity before or after diagnosis associates with reduced disease-specific mortality. Previous mechanistic studies indicate that components of innate immunity can mediate an inhibitory effect of physical activity on several types of tumor. However, in breast cancer specifically, the myeloid compartment of innate immunity is thought to exhibit high propensity for an immunosuppressive role that obstructs anti-tumor immunity. Thus, we tested the notion that greater physical activity alters mononuclear phagocytes in mammary tissue when inhibiting nascent tumor in a murine model of breast cancer. To model greater physical activity, we placed an angled running wheel in each mouse’s home cage for two weeks before tumor engraftment with EO771 mammary cancer cells that express luciferase for bioluminescent detection. Fully immunocompetent mice and mice with compromised adaptive immunity showed significantly less mammary tumor signal when given access to running wheels, although the effect size was smaller in this latter group. To investigate the role of the myeloid compartment, mononuclear phagocytes were ablated by systemic injection of clodronate liposomes at 24 h before tumor engraftment and again at the time of tumor engraftment, and this treatment reversed the inhibition in wheel running mice. However, clodronate also inhibited mammary tumor signal in sedentary mice, in conjunction with an expected decrease in gene and protein expression of the myeloid antigen, F4/80 (Adgre1), in mammary tissue. Whole transcriptome digital cytometry with CIBERSORTx was used to analyze myeloid cell populations in mammary tissue following voluntary wheel running and clodronate treatment, and this approach found significant changes in macrophage and monocyte populations. In exploratory analyses, whole transcriptome composite scores for monocytic myeloid-derived suppressor cell (M-MDSC), macrophage lactate timer, and inflammation resolution gene expression programs were significantly altered. Altogether, the results support the hypothesis that physical activity inhibits nascent mammary tumor growth by enhancing the anti-tumor potential of mononuclear phagocytes in mammary tissue.

## Introduction

By the end of the current year, it is estimated that well over a quarter million new cases of invasive breast cancer will be diagnosed in the US, being by far the most common type of (non-skin) cancer in women (Siegel, Miller & Jemal, 2020). However, several studies have found that this risk for breast cancer is significantly reduced in persons who engage in greater amounts of physical activity (Moore et al., 2016). Further analysis of molecular subtypes in breast cancer suggests the association holds whether tumors are hormone receptor (ER/PR) positive or negative (Ma et al., 2016). Moreover, meta-analyses of breast cancer survival studies indicate that greater physical activity either before or after diagnosis associates with reduced disease-specific mortality, with evidence of a dose–response effect (Ballard-Barbash et al., 2012; Friedenreich et al., 2016). Given the enormity of the findings, the World Health Organization’s IARC (International Agency for Research on Cancer) has assigned its strongest evidence designation to physical activity as a preventative factor in breast cancer (Vainio & Bianchini, 2002).

Several broad pathways have been proposed to mediate the link between physical activity and cancer generally, including enhancement of immune parameters (McTiernan, 2008; Koelwyn et al., 2017; Hojman et al., 2018). More recently, mechanistic studies in mice indicate that components of innate immunity can mediate an inhibitory effect of physical activity on several types of tumor (Pedersen et al., 2016). However, in breast cancer specifically, the myeloid compartment of innate immunity is thought to exhibit high propensity for an immunosuppressive role that obstructs anti-tumor immunity (Kim et al., 2019). Subsequently, members of the mononuclear phagocyte system, i.e., monocytes, macrophages, and dendritic cells (Guilliams et al., 2014), are found to exhibit variable roles in breast cancer. Monocytes have been found to exhibit both pro-tumoral and anti-tumoral roles (Olingy, Dinh & Hedrick, 2019). Similarly, macrophages can exhibit a phenotype that varies from a broadly defined M2-like macrophage that suppresses immunity to an equally broadly defined M1-like macrophage that supports anti-tumor actions (Mantovani et al., 2017). Dendritic cells (DCs) indicate a worse prognosis if they manifest an immature phenotype in primary breast tumors or sentinel lymph nodes, likely because of the role mature DCs play in antigen presentation for adaptive immunity (Gibert-Ramos et al., 2019).

It seems reasonable that physical activity’s inhibitory effect on breast cancer incidence would require promotion of a myeloid compartment in mammary tissue that tilts away from immunosuppression and/or toward anti-tumor immunity. Immunosuppression is a correlate of inflammation resolution, where leukocyte recruitment to an inflammatory site desists and regulatory cells are activated (Ortega-Gómez, Perretti & Soehnlein, 2013). Such resolution is not a passive process but involves active pro-resolving programs, of which the transcriptomes for such programs are becoming clearer. For example, Zhang et al. (2019a) found that an initial M1-promoting pro-inflammatory stimulus sets in motion a slow accumulation of intracellular lactate that epigenetically up-regulates gene expression to turn the macrophage toward a more homeostatic phenotype to facilitate tissue repair, which they refer to as a “lactate timer”. Additionally, Kong et al. (2020) found that mononuclear phagocytes in a model of sterile inflammation contribute to an up-regulated tissue transcriptome that associates with the resolution of the inflammatory event.

Such mononuclear phagocytes are affected by physical activity. Acute physical exercise rapidly increases the number of monocytes in the bloodstream (Walsh et al., 2011), but the non-classical phenotype goes significantly below baseline by 1 h after exercise (Gabriel et al., 1994; Shantsila et al., 2012), which has been interpreted as a likely extravasation into tissue following re-attachment to the endothelium (Gabriel et al., 1994). This significant dip occurs despite the fact that acute exercise induces a greater proportion of these non-classical monocytes into the bloodstream vs. classical monocytes (Gabriel et al., 1994; Steppich et al., 2000; Heimbeck et al., 2010; Radom-Aizik et al., 2014). Analysis of mononuclear phagocytes following exercise has found variable levels of down-regulated gene expression for the murine myeloid antigen, F4/80 (*Adgre1*) (Gordon et al., 2011) in adipose tissue (Kawanishi et al., 2010) and intestinal mucosal lining (McClellan et al., 2014), but no change in the number of F4/80^+^ macrophages in the peritoneum following exercise (Kizaki et al., 2008). Several studies have found that exercise augments the M1-like response of LPS-stimulated peritoneal macrophages (Sugiura et al., 2002; Kizaki et al., 2008; Abdalla et al., 2014), although these studies did not determine whether M2 capacity is also augmented following exercise.

Fewer studies have directly examined the role of mononuclear phagocytes in mammary tissues and/or their tumors following physical activity. In the highly metastatic 4T1 syngeneic breast cancer model, exercise did not significantly alter macrophage number in mammary tumor but did reduce MHCII expression on macrophages (Bianco et al., 2017), which could be interpreted as a shift toward the M2-like phenotype (Cao et al., 1989). Subsequently, tumor DCs were significantly reduced by exercise, although MHCII and co-stimulatory antigens CD80 and CD86 were nonetheless up-regulated on such cells. Although not examining mononuclear phagocytes, a separate laboratory using the same 4T1 breast cancer model found that exercise also significantly reduced myeloid derived suppressor cells (MDSCs) in the tumor (Wennerberg et al., 2020). In both studies with the 4T1 model, these exercise-induced alterations in myeloid cell subsets associated with inhibition of mammary tumor. However, direct mediation by these cell populations in physical activity’s effect on mammary tumor was not tested.

In the present study, we tested the hypothesis that greater physical activity inhibits nascent mammary tumor growth by altering mononuclear phagocytes in mammary tissue. To accomplish this, we conducted a series of experiments where mice were given access to running wheels in their home cages and mononuclear phagocytes were ablated with clodronate liposomes (Weisser, Rooijen & Sly, 2012) before subsequent tumor engraftment. We found that inhibition of mononuclear phagocytes altered the expression of F4/80 (Adgre1) in mammary tissue and reversed the inhibitory effect of voluntary wheel running on mammary tumor growth. Digital cytometry further determined specific mononuclear phagocyte subsets in mammary tissue that were altered in running mice, and additional whole transcriptome exploratory analyses suggested that running-induced modulation of mononuclear phagocytes may be associated with inhibition of an inflammation resolution program.

## Materials & Methods

### Animals

Female C57BL/6J mice were obtained from The Jackson Laboratory. Severe combined immunocompromised CB17/Icr-*Prkdc*^*scid*^ mice were obtained from Charles River Laboratories. Mice were 7–8 weeks old, housed under BSL2 barrier conditions on an individually ventilated cage (IVC) rack in dual filter disposable cages (Innovive, Inc.), with corn cob bedding, nesting tissue for enrichment, and ad libitum access to food and water on a 12:12 light:dark cycle at 22 °C. At the conclusion of each experiment, all mice were euthanized via anesthetization with isoflurane followed by cervical dislocation. Mice were euthanized prior to study completion if they met any of the following criteria: (a) Body Condition Score of 2 or less (Ullman-Cullere & Foltz, 1999), (b) ruffled fur, squinted eyes, dull mentation, or respiratory difficulty, or (c) lameness or impaired mobility. No mice met the criteria for euthanasia prior to study completion. All procedures were carried out under protocols approved by the Institutional Animal Use and Care Committee of the University of California, Los Angeles (#2007-155).

### Voluntary wheel running

To model voluntary physical activity, we placed an angled running wheel-hub apparatus in each mouse’s home cage (Fast-Trac Mouse Igloo®, Bio-Serve, Flemington, NJ). Control mice received the hub only. Although effects of exercise on myeloid cell function have been reported after just 6 days of daily running (e.g., greater peritoneal macrophage tumor cytotoxicity; Murphy et al., 2004), in the current study mice were given access to the wheel-hub for two weeks prior to tumor engraftment. This time point was chosen because we wanted to increase the likelihood that animals would provide their strongest running sessions around the time of tumor engraftment, and previous research finds average daily running time and running distance begin to plateau after two weeks in this voluntary wheel running paradigm (De Bono et al., 2006). Also, physiological changes denoting training effects have been reported at this time point, which include changes in heart rate acceleration at the outset of running and buffered increases in systolic blood pressure at the outset of cage activity (Adlam et al., 2011). In each of three independent experiments for both C57BL/6J and SCID mouse paradigms, *n* = 5–6 mice per group.

### Clodronate liposomes

To ablate mononuclear phagocytes (i.e., monocytes, macrophages, dendritic cells), a suspension of clodronate liposomes was injected intravenously at 100uL/10g of body weight (Liposoma Research via Cedarlane Biologicals, #CP-005-005). Injections were performed 24 h before tumor engraftment and again at the time of tumor engraftment. Control mice were injected with PBS vehicle liposomes. In each of three independent experiments where voluntary wheel running was crossed with clodronate treatment (2 × 2 factorial design), *n* = 5–6 mice per group.

### Lentiviral vector

To produce lentiviral vector containing firefly luciferase for mammary cancer cell transduction, 293T cells (ATCC® CRL3216™) were cultured in IMDM (Sigma-Aldrich, St. Louis, MO) containing 10% FBS (Sigma-Aldrich) and antibiotics. Plasmid encoding FUG2ALucW was generated by replacing EGFP sequence of FUGW (Lois et al., 2002) with the sequence of the fusion protein (G2ALuc; Ibrahimi et al., 2009) of EGFP, self-cleaving T2A sequence derived from Thosea asigna virus, and firefly luciferase. This vector is resistant to silencing in vivo, so that luciferase expression level does not change in response to alterations in cell environment circumstances. G2ALuc-expressing lentiviral vector pseudotyped with VSV-G was produced by transfecting 293T cells with VSV-G, packaging plasmid PAX2 (Addgene, Cambridge, MA), and a lentiviral vector (FUG2ALucW) using TransIT LT1 (MirusBio, Madison, WI) (Chua et al., 2019) (Fig. S1). The supernatant was harvested and filtered 72 h post transfection for subsequent transduction of the EO771 mammary cancer cell line (CH3 Biosystems, LLC).

### Orthotopic breast cancer model

Modeling was conducted as previously described in Lamkin et al. (2015). Briefly, transduced EO771 cells expressing firefly luciferase were cultured in RPMI-1640 with L-glutamine (Cellgro-Corning, Inc., #10-040-CV), supplemented with 10% FBS (Atlanta Biologicals, #S11550H), at 37 °C, in a 5% CO_2_ atmosphere. After harvesting, tumor cells (1 × 10^5^) were injected into the left fourth mammary fat pad (Day 0). Nascent mammary tumor signal was then measured in live mice by repeated noninvasive optical imaging of tumor-specific luciferase activity using the IVIS Lumina II system running Living Image 4.7 software (PerkinElmer). After anesthetization with 2% isoflurane and intravenous injection of 150 mg/kg luciferin, mice were photographed under bright-field illumination and images were overlaid with luminescence data gathered over the maximum exposure period without pixel saturation (1–60 s). Mammary tumor signal was measured by triplicate determination at each time point of total bioluminescence in a region of interest (ROI) of constant size. For two-group experiments that examined the effect of wheel running, tumor signal was measured on Day 0, Day1, and Day 7. For factorial experiments that examined the effects of both wheel running and clodronate, tumor signal was measured only on Day 0 and Day 1 to enable early mammary tissue collection.

### RT-qPCR

To quantify gene expression from mammary tissue, total RNA from RLT lysates was extracted (Qiagen RNeasy Mini Kit, #74104), cleared of contaminating DNA with on-column DNase digestion (Qiagen RNase-Free DNase Set, #79254), and quantified by spectrophotometry (NanoDrop ND-1000; Thermo Scientific). As previously described in Lamkin et al. (2019), gene transcripts were examined by RT-qPCR with the CFX96 Touch Real-Time PCR Detection System (Bio-Rad), using one-step assay reagents (Qiagen Quantitect Probe RT-PCR, #204443) and TaqMan Gene Expression Assay primer-probes for mouse myeloid antigen F4/80 (Gordon et al., 2011), i.e., *Adgre1* (Mm00802529_m1; Thermo Fisher). Following reverse transcription of RNA template for 30 min at 50 °C, resulting product underwent an initial activation step at 94 °C for 15 min followed by 50 amplification cycles of 15 s of strand separation at 94 °C and 60 s of annealing and extension at 60 °C. Triplicate determinations of each biological replicate were quantified by threshold cycle analysis of FAM fluorescence intensity using CFX Manager software (Bio-Rad), normalized to values of beta-actin mRNA amplified in parallel (#Mm00607939_s1).

### Immunofluorescent microscopy

For analysis of F4/80 protein expression in mammary tissue, whole mammary tissue fat pads were dissected and fixed in 10% neutral buffered formalin for 24 h. Tissues were then rinsed in running water for 5 min and stored in reagent grade alcohol until undergoing paraffin embedding and cutting into 4 µm sections for immunofluorescent staining. Sections were deparaffinized in two separate xylene preparations for 5 min each, rehydrated through 1 min incubations in ethanol of decreasing concentration (100%, 90%, 70%, 30%), and incubated in distilled water for at least 1 min before undergoing heat-induced epitope retrieval with a steamer in ∼95 °C citrate buffer (10mM Citric Acid, 0.05% Tween20, pH 6.0) for 20 min. Sections were then submerged in room temperature water to cool down for at least 1 min before being incubated in a 37 °C solution of trypsin-EDTA (0.025%–0.002%) in PBS for 1 min. Sections were then rinsed in two changes of PBS for 2.5 min each. Sections were then incubated with blocking solution (#NB309; Innovex) for 30 min before overnight incubation at 4 °C with rat monoclonal antibodies against mouse F4/80 (BM8 clone; Thermo Fisher, # 14-4801-82) at 1/50 dilution. Following washing, slides were then incubated with goat anti-rat IgG secondary antibodies conjugated to Alexa 594 fluorophores (# A-11007; Thermo Fisher,) at 1/500 dilution for 2 h, washed, and incubated with DAPI nuclear counter stain solution (Molecular Probes #R37606). A negative control slide was prepared for each specimen by removing the primary antibody from the staining process. As previously described in Lamkin et al. (2019), coverslips were then mounted onto sections using anti-fade fluorescent mounting media (#S302380-2; Agilent). Fluorescence was visualized with a Leica DM6000 B upright microscope and fluorescence filters, using a 20 × objective with NA = 0.8 and flat field optical and fluorite aberration correction. Digital images of each specimen were acquired with a Leica DFC365 FX monochromatic camera and software, with gain and exposure time held constant across conditions of a given experiment. To quantify the relative amount of F4/80 protein, 18 random fluorescent images of the specimen were acquired as tagged image format files to accomplish in situ assay via pixel threshold analysis with ImageJ software (Schneider, Rasband & Eliceiri, 2012; Hartig, 2013).

### Whole transcriptome digital cytometry and transcriptome representation analysis (TRA)

As previously described in Lamkin et al. (2019), total RNA (500 ng per sample) was converted to cDNA (Lexogen QuantSeq 3′FWD) and sequenced using an Illumina HiSeq 4000 instrument in the UCLA Neuroscience Genomics Core Laboratory. Low-level sequencing data was mapped to the RefSeq mouse genome sequence and normalized to counts per million mapped reads (CPM) using the STAR aligner. Transcriptome data from the present study are deposited in the National Center for Biotechnology Information Gene Expression Omnibus (GSE150620).

To estimate the percentage of myeloid cell subsets in each sample, the sample’s transcriptome data were deconvolved with the CIBERSORTx support vector regression machine learning approach (Newman et al., 2015; Newman et al., 2019), with batch correction enabled for RNAseq data, in conjunction with the ImmuCC input matrix of murine reference gene expression signatures for 25 immune cell types (Chen et al., 2017), which include reference data for neutrophils, monocytes, resting macrophages (M0), M1-like and M2-like macrophages, and immature and activated DCs (Data S2).

To test for group differences in the prevalence of transcriptomes for monocytic and granulocytic myeloid-derived suppressor cells (M-MDSC and G-MDSC) described by Alshetaiwi et al. (2020), the macrophage lactate timer described by Zhang et al. (2019a), and the inflammation resolution program described by Kong et al., (2020), we conducted a transcriptome representation analysis (TRA) (Powell et al., 2013). For each of the TRAs, a transcriptome’s composite score was computed for a given subject by calculating the mean expression value, in that subject, of all genes specified in the reference set of the transcriptome in question. Before this mean was calculated for a given subject, all expression values across subjects for a given gene were z-score-transformed to move all genes onto the same scale. The reference sets for M-MDSCs and G-MDSCs were composed of genes from CD45^+^CD11b^+^Gr1^+^ phenotypic MDSC cells that best identified functional (i.e., immunosuppressive) MDSCs vs. non-functional MDSCs of both the monocytic and granulocytic lineages, as evidenced by >50% differential expression in the functional cells (Alshetaiwi et al., 2020) (Data S3). Reference set for active resolution of inflammation was composed of genes that were up-regulated by >75% at the resolution of a sterile inflammatory event vs. its peak (Kong et al., 2020) (Data S4). Reference set for the macrophage lactate timer was composed of genes in macrophages that were up-regulated by >100% at 24 h following an M1-promoting pro-inflammatory stimulus in vitro (i.e., LPS) (Zhang et al., 2019a) (Data S5). These up-regulated genes appear to mark an inflammatory resolution phase and largely coincided with genes regulated by histone lactylation in macrophages that were representative of M2-like polarization (Zhang et al., 2019a).

### Statistical analysis

**Figure 1:**
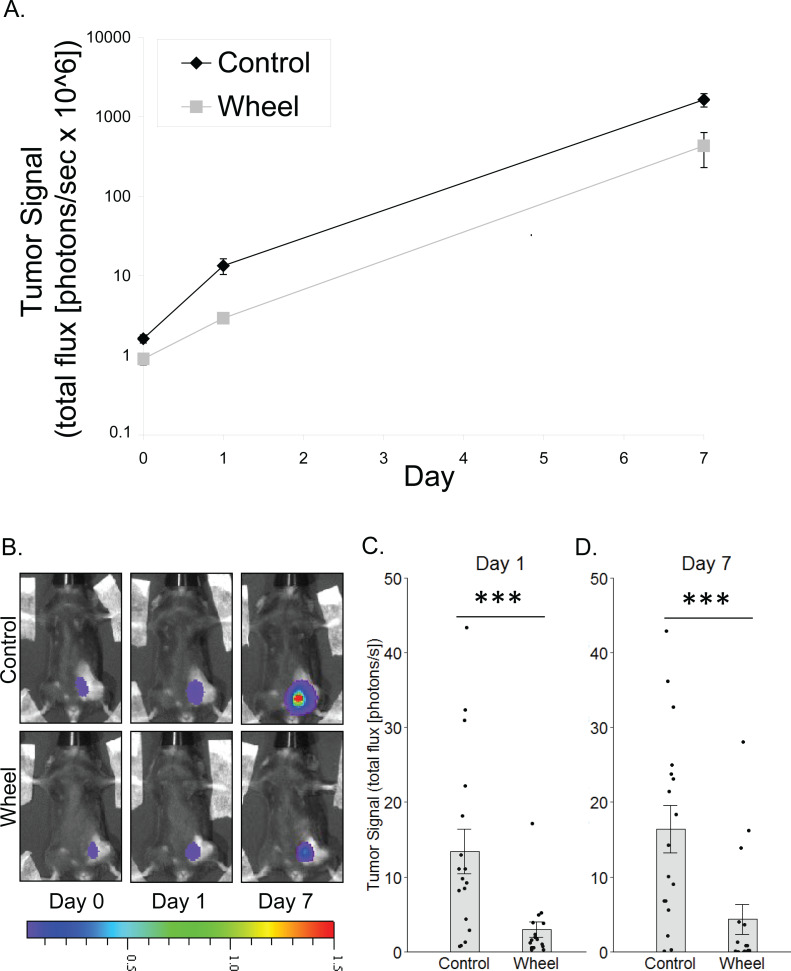
Effect of voluntary wheel running on mammary tumor in the C57BL/6-EO771 model of breast cancer. (A) Trajectory of tumor signal (mean ± SEM on logarithmic scale) from engraftment on Day 0 to Day 1 and Day 7 in control mice and voluntary wheel running mice. (B) Representative images of bioluminescent tumor signal in C57BL/6 mice; radiance color scale is photos/s/cm^2^/sr × 10^7^; radiance scale minimum is one order of magnitude lower for Day 0 and Day 1. (C–D) Mean ± SEM and individual data points of three independent experiments with *n* = 4–6 mice per group per experiment for (C) Day 1 (×10^6^) and (D) Day 7 (×10^8^). ***P < 0.001 for bars under horizontal line.

**Figure 2:**
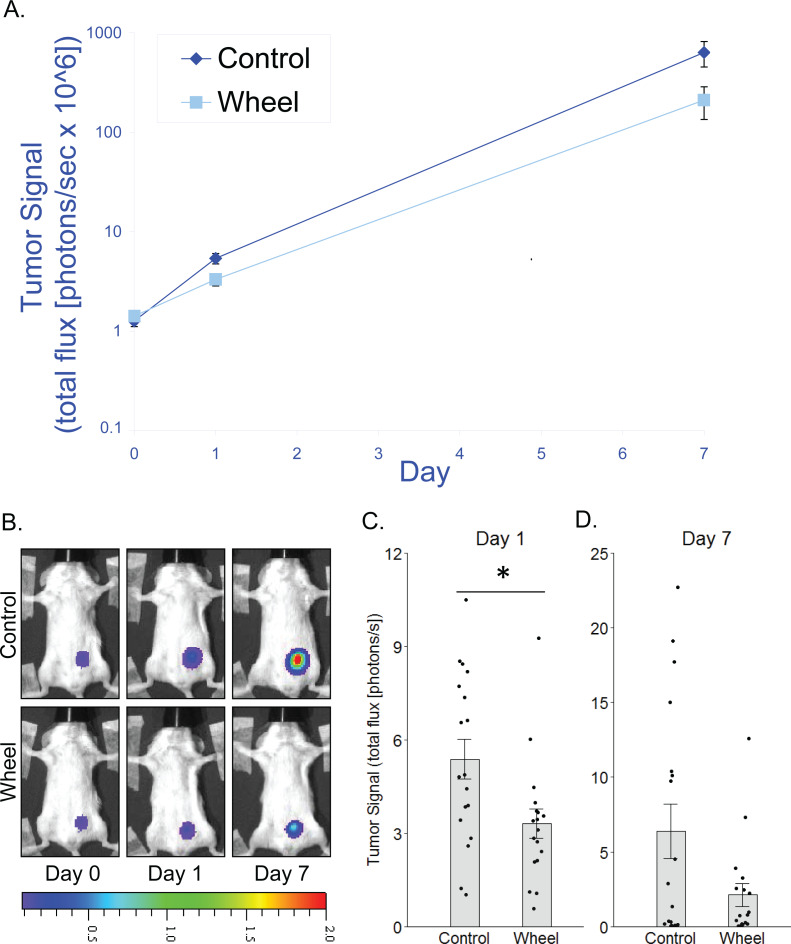
Effect of voluntary wheel running on EO771 mammary cancer in SCID mice. (A) Trajectory of tumor signal (mean ± SEM on logarithmic scale) from engraftment on Day 0 to Day 1 and Day 7 in control mice and voluntary wheel running mice. (B) Representative images of bioluminescent tumor signal in SCID mice; radiance color scale is photos/s/cm^2^/sr × 10^7^; radiance scale minimum is one order of magnitude lower for Day 0 and Day 1. (C–D) Mean ± SEM and individual data points of three independent experiments with *n* = 6 mice per group per experiment for (C) Day 1 (×10^6^) and (D) Day 7 (×10^8^). *P < 0.05 for bars under horizontal line.

**Figure 3:**
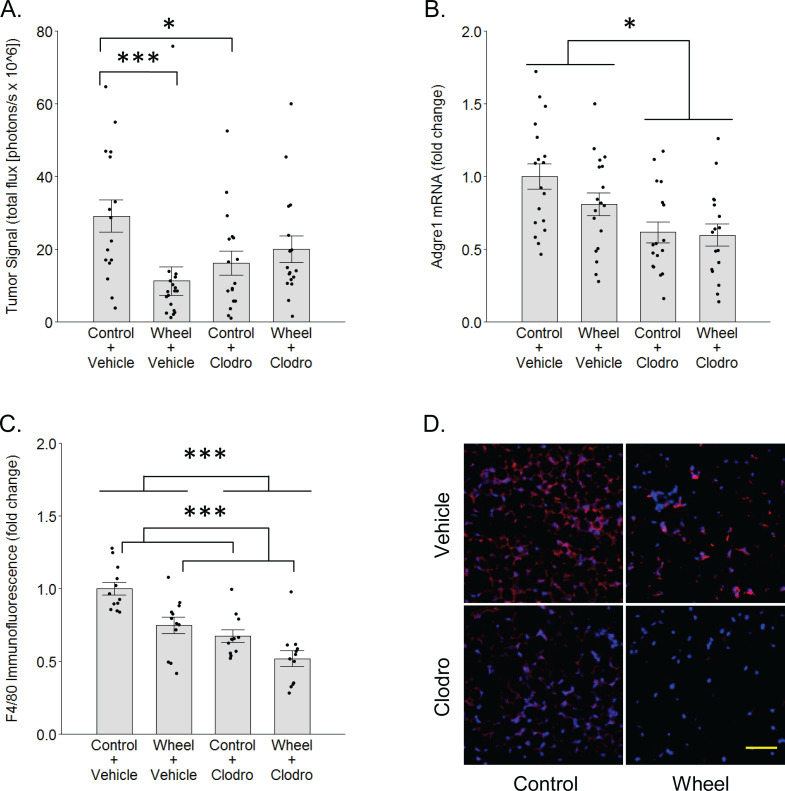
Effects of voluntary wheel running × clodronate treatment in the immunocompetent C57BL/6-EO771 model of breast cancer. (A) Effects on tumor signal in mammary tissue at Day 1. Mean ± SEM and individual data points of three independent experiments, with *n* = 5–6 mice per group per experiment. (B) Concurrent effects on *Adgre1* gene expression in mammary tissue at Day 1. Mean ± SEM and individual data points of three independent experiments, with *n* = 5–6 mice per group per experiment. (C) Concurrent effects on F4/80 protein expression in mammary tissue at Day 1. Mean ± SEM and individual data points of two independent experiments, with *n* = 5–6 mice per group per experiment. (D) Matrix of representative images of F4/80 immunofluorescence (red) in mammary tissue sections with nuclei (blue) at Day 1, scale bar 50 µm. ***P < 0.001, *P < 0.05 for bars or groups of bars under horizontal lines. Clodro: Clodronate.

**Figure 4:**
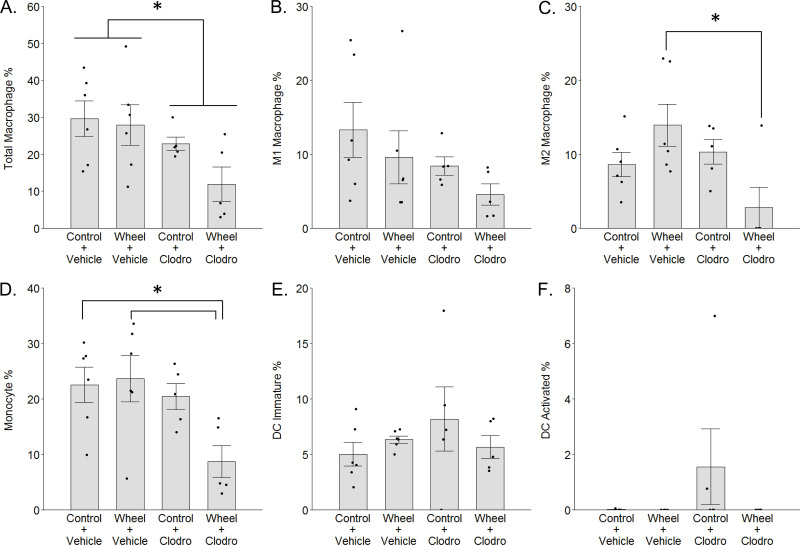
Mononuclear phagocyte proportions in mammary tissue as estimated by CIBERSORTx with the ImmuCC reference gene set at Day 1 in the immunocompetent C57BL/6-EO771 model of breast cancer. Mean ± SEM and individual data points, with *n* = 5–6 mice per group. In (F), only three subjects exhibited non-zero values. *P < 0.05 for bars or groups of bars under horizontal lines. Clodro: Clodronate.

**Figure 5:**
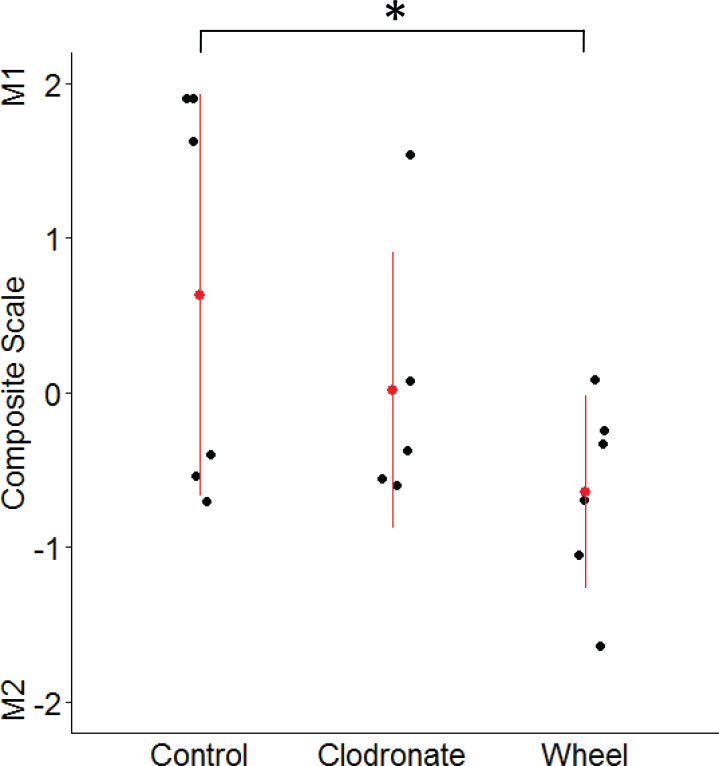
Mammary macrophage phenotype among groups from the wheel running × clodronate factorial paradigm in the immunocompetent C57BL/6-EO771 model of breast cancer. The multivariate composite variable that maximally discriminated among voluntary wheel running-only mice (Wheel), clodronate-only mice (Clodronate), and control mice (Control) on measures of M1-like and M2-like macrophage percentages at Day 1 was re-constructed. Positive side of the composite variable is defined by relatively more M1-like and less M2-like macrophages while the negative side is defined by less M1-like and more M2-like. Mean ± SD indicated by red point with vertical range line; individual data points in black, with *n* = 5–6 mice per group. *P < 0.05 for Wheel vs. Control.

**Figure 6:**
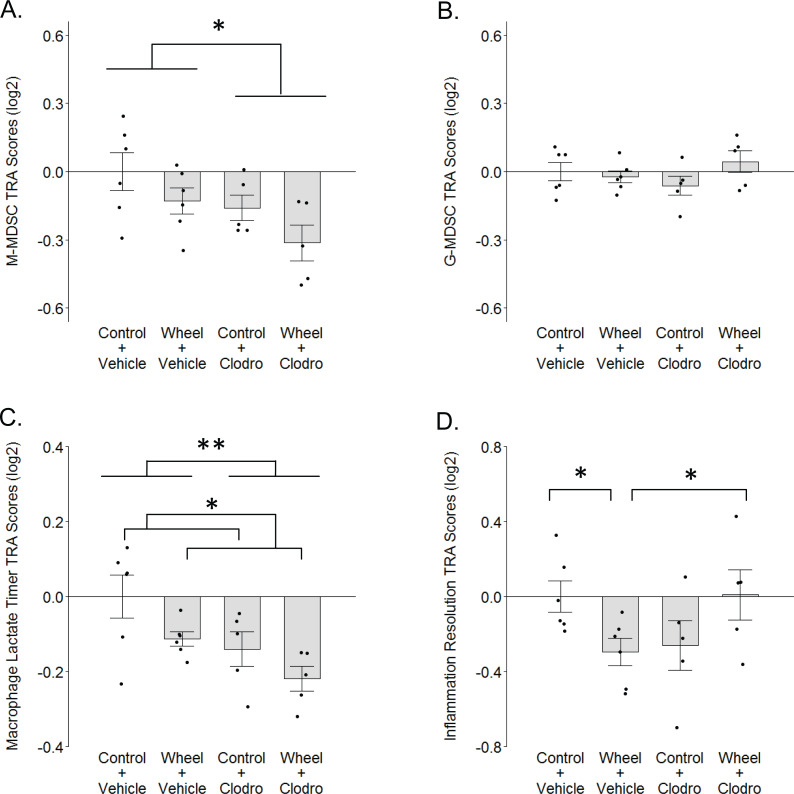
TRA composite scores in the immunocompetent C57BL/6-EO771 model of breast cancer as a function of voluntary wheel running × clodronate treatment at Day 1. (A) Monocytic myeloid-derived suppressor cell (M-MDSC) transcriptome. (B) Granulocytic myeloid-derived suppressor cell (G-MDSC) transcriptome. (C) Macrophage lactate timer transcriptome. (D) Inflammation resolution transcriptome. Mean ± SEM and individual data points, with *n* = 5–6 mice per group. Scores on log2 scale centered at zero. See text for references for genes sets. Defining genes for each transcriptome are listed in Data S3–S5. ** *P* < 0.01, * *P* < 0.05 for bars or groups of bars under horizontal lines. Clodro: Clodronate.

Analyses were conducted in the R statistical environment, version 3.6.1 (R Core Team, 2019), with additional packages cited below. All distributions were examined for outliers and transformations were applied to normalize distributions where necessary. For Figs. 1 and 2, Student’s *t*-test was used to analyze the effect of voluntary wheel running on tumor signal at each time point in the context of a repeated measures univariate analysis of variance (Singmann et al., 2019). For Figs. 3 and 4, factorial analysis of variance was used to examine the effects of voluntary wheel running and clodronate on tumor signal, *Adgre1* gene expression, F4/80 (Adgre1) protein expression, and CIBERSORTx estimates of mononuclear phagocyte proportions in mammary tissue, with Tukey’s adjustment for multiple comparisons. For Fig. 5, discriminant function loadings for the M1-like and M2-like variables from a multivariate analysis of variance (Friendly & Fox, 2017) were used to re-construct the composite variable that maximally discriminated among voluntary wheel-running mice, clodronate mice, and control mice. Fisher’s univariate procedure was then used to conduct pairwise comparisons to test for differences in macrophage polarity phenotype as captured in the composite variable. Given that the discriminant function loadings are based on multivariate normality, transformed data were examined to ensure group univariate distributions were approximately symmetrical (skewness coefficients between −0.55 and +0.55) (Meyer et al., 2019) and did not significantly deviate from normality on the Shapiro–Wilk test. The Mahalanobis distance measure (*D*^2^) was used to assess presence of multivariate outliers and found that all *D*^2^ values were within 3 standard deviations of the mean on the corresponding chi-square distribution. Box’s M-test was used to ensure covariance matrices were not significantly different (Da Silva, Malafaia & Menezes, 2017). For Fig. 6, composite scores among groups were analyzed for each TRA with factorial analysis of variance to examine the effects of voluntary wheel running and clodronate on each transcriptome of interest, with Tukey’s adjustment for multiple comparisons.

## Results

### Inhibitory effect of voluntary wheel running on tumor growth

To model the link between greater physical activity and lower breast cancer incidence, we determined the extent to which physical activity could inhibit nascent mammary tumor growth in mice by using in vivo bioluminescent imaging of the mammary cancer cell line, EO771, which is syngeneic to the immunocompetent C57BL/6 mouse strain. There was a significant group × time interaction effect on mammary tumor signal (*P* = 0.0006; Fig. 1). Mice that were given access to running wheels showed significantly less tumor signal than control mice at Day 1 and at one week after cancer engraftment (mean inhibition = 78% ± 3%, *P* = 0.0005; 74% ± 12%, *P* = 0.0004, respectively).

### Adaptive immunity is not necessary for an inhibitory effect of wheel running on tumor growth

To determine whether adaptive immunity is necessary for the effect of running wheel access on EO771 tumor signal, we injected EO771 cells into the mammary fat pad of severe combined immunodeficient (SCID) mice (i.e., CB17/Icr-*Prkdc*^scid^) that lack functional T cells and B cells. Consistent with results from fully immunocompetent mice, SCID mice that were given access to running wheels also showed significantly less tumor signal than control mice by Day 1, although the effect size was smaller (mean inhibition = 38% ± 9%, *P* = 0.019; Fig. 2). Consequently, the effect was no longer significant by Day 7 (*P* = .13; Fig. 2). These results suggested that innate immune system components alone can mediate an inhibitory effect of physical activity on EO771 growth, although adaptive immune mechanisms may additionally contribute to the overall effect. Thus, to further define the role of mononuclear phagocytes in the inhibitory effect of voluntary wheel running, we returned to the fully immunocompetent EO771 model to conduct additional studies with mononuclear phagocyte ablation.

### Reversal of wheel running’s inhibitory effect by mononuclear phagocyte ablation

To determine whether mononuclear phagocytes are necessary for the inhibitory effect of wheel running on tumor growth, we treated immunocompetent C57BL/6 mice with mononuclear phagocyte-ablating clodronate liposomes one day before and again on the day of cancer cell engraftment. There was a significant group × time interaction effect (*P* = 0.014) on mammary tumor signal. As seen in Fig. 3, voluntary wheel running significantly decreased tumor signal compared to sedentary controls by Day 1 (mean inhibition: 67% ± 11%, *P* = 0.0004), but clodronate reversed this inhibition to the point that voluntary wheel running no longer exerted a significant effect (mean inhibition: 42% ± 10%, *P* = 0.26). Despite this reversal in the voluntary wheel running group, clodronate by itself inhibited tumor signal in the sedentary group (*P* = 0.028). Further examination of clodronate’s effect found that it significantly reduced gene expression of the mononuclear phagocyte marker *Adgre1* in the mammary tissues of both sedentary and wheel running mice as expected (mean inhibition: 39% ± 7%, *P* = 0.0005 for main effect; Fig. 3B). Mice in the wheel running-only group also appeared to exhibit a decrease in *Adgre1* expression, albeit smaller, but neither the main effect of running nor the interaction with clodronate were significant (*P* = 0.22 and *P* = 0.43, respectively; the simplest unadjusted two-group comparison with control mice also failed to reach significance with *P* = 0.11). However, protein expression results followed a similar pattern where both clodronate and voluntary wheel running significantly reduced F4/80 immunofluorescence (mean inhibition: 32% ± 6%, *P* < 0.0001 and 25% ± 6%, *P* = 0.0002, for main effect, respectively; Figs. 3C–3D). These results suggested that physical activity may alter the anti-tumor potential of the myeloid compartment in mammary tissue by modulating one or more mononuclear phagocyte populations. To further address this possibility, we conducted genome-wide transcriptional profiling to examine the frequency of mononuclear phagocyte populations in mammary tissue.

### Whole transcriptome determination of mononuclear phagocytes in mammary tissue following wheel running and clodronate treatment

To investigate the potential role of mononuclear phagocyte modulation in the inhibitory effect of wheel running on tumor growth, we conducted digital cytometry with the CIBERSORTx platform and the mouse signature gene matrix file, ImmuCC, to calculate the relative percentages of total macrophages, M1-like and M2-like macrophages, monocytes, immature dendritic cells (DCs), and activated DCs from mammary tissue transcriptome data. In accord with F4/80 results, clodronate significantly decreased total macrophage percentage (mean inhibition: 40% ± 17%, *P* = 0.024 for main effect; Fig. 4A), although the effect appeared somewhat stronger among clodronate mice that were also wheel runners (*P* = 0.19 for running main effect; *P* = .33 for interaction effect). M1-like macrophage percentages followed a similar pattern (Fig. 4B), but the effects did not reach statistical significance (*P* = 0.09 for running main effect; *P* = 0.11 for clodronate main effect; *P* = .66 for interaction effect). In contrast, M2-like macrophage percentages exhibited a significant interaction effect (*P* = 0.013; Fig. 4C), where substantially higher numbers in the wheel running-only group were significantly reversed by clodronate (mean inhibition: 80% ± 17%, *P* = 0.016) (*P* = 0.64 for running main effect; *P* = 0.06 for clodronate main effect). Similarly, monocyte percentages also exhibited a marginal interaction effect (*P* = 0.07; Fig. 4D), where higher numbers in the wheel running-only group were significantly reversed by clodronate in wheel running mice (mean inhibition: 63% ± 15%, *P* = 0.026) (*P* = 0.13 for running main effect; *P* = 0.020 for clodronate main effect). Immature DCs were detected in all groups, but there were no significant differences (Fig. 4E; *P* = 0.96 for running main effect; *P* = 0.55 for clodronate main effect; *P* = .21 for interaction effect). In contrast, activated DCs were absent in nearly all the samples (Fig. 4F), making quantitative analysis infeasible. Because neutrophils are involved in exercise and cancer (Peake, 2002; Zielinski et al., 2004), we examined their percentages but found that they also exhibited low numbers and no significant effects of exercise or clodronate (*P* values > 0.49; Fig. S2).

Given the unexpected finding of greater M2-like macrophage prevalence in the groups with lower tumor signal, as well as the inter-relatedness of these outcome variables, we examined them simultaneously by using the discriminant function loadings from a multivariate analysis of variance to re-construct the composite variable that maximally discriminates among voluntary wheel-running mice, clodronate mice, and control mice. The resulting standardized coefficients indicated that M1 macrophages loaded positively onto the composite variable (0.62), while M2 macrophages loaded negatively (−0.92). Thus, the composite variable was defined by relatively more M1-like and less M2-like macrophages on the positive side of the variable and less M1-like and more M2-like macrophages on the negative side. As seen in Fig. 5, voluntary wheel running mice significantly differed in macrophage phenotype from control mice (*P* = 0.042). Running mice had relatively less M1-like and more M2-like macrophages by Day 1 when tumor had been significantly inhibited, in accord with the univariate M1-like and M2-like macrophage findings above. In contrast, clodronate mice exhibited no significant difference in macrophage phenotype versus controls (*P* = 0.32) and manifested a composite variable score that was closer to zero, indicative of relatively more equal proportions of M1-like and M2-like macrophages. Thus, despite similar levels of tumor inhibition in the wheel running-only and clodronate-only conditions (see Fig. 3), mammary tissue macrophage phenotype appears to be less similar between these two groups.

### Whole transcriptome exploratory analyses

To further investigate the finding of relatively higher M2-like macrophage values in the wheel running-only group, we considered the possibility that myeloid-derived suppressor cells (MDSCs) might also be higher in this group vs. wheel running mice receiving clodronate. Crosstalk between MDSCs and macrophages can induce the latter toward a phenotype in mammary cancer that can be construed as M2-like (Sinha et al., 2007). Thus, we calculated transcriptome representation analysis (TRA) (Powell et al., 2013) composite scores for both monocytic (M-MDSC) and granulocytic (G-MDSC) myeloid-derived suppressor cell phenotypes from the mammary tissue transcriptome data. Clodronate exerted a significant inhibitory effect on M-MDSC TRA scores (*P* = 0.025 for main effect; Fig. 6A) and running exerted a marginally significant inhibitory effect (*P* = 0.06 for main effect of running), but there was no significant interaction effect (*P* = 0.86). G-MDSCs exhibited smaller TRA scores with no significant differences among groups (Fig. 6B; *P* = 0.33 for running main effect; *P* = 0.96 for clodronate main effect; *P* = .12 for interaction effect). Thus, there is some indication that M-MDSCs are higher in wheel running-only mice than in wheel runners receiving clodronate, but overall it would appear M-MDSCs are reduced by both running and clodronate, with additive effect.

We also considered the possibility that the recently characterized “lactate timer” in macrophages might contribute to the higher M2-like numbers in wheel running-only mice, where an initial M1-promoting pro-inflammatory stimulus also sets in motion a slow accumulation of intracellular lactate that epigenetically alters gene expression to turn the macrophage toward a more homeostatic phenotype (Zhang et al., 2019a; Murray, 2020). TRA scores for the macrophage lactate timer exhibited significant effects but in directions opposite of what might be expected, as both running and clodronate caused inhibitory effects on lactate timer gene expression (Fig. 6C; *P* = 0.032 for running main effect; *P* = 0.009 for clodronate main effect; *P* = .68 for interaction effect). Thus, it would appear that the higher M2-like numbers in wheel running-only mice are not a result of the lactate timer directing macrophages toward a more homeostatic phenotype.

M2-like phenotype in wheel runners notwithstanding, the foregoing result of a relatively non-homeostatic macrophage in this group led us to further consider the hypothesis that wheel runners exhibit a broader non-resolved inflammatory state. Not simply representative of one macrophage phenotype, the up-regulated gene set identified by Kong et al. (2020) that associates with resolution of a sterile inflammatory event is constituted by multiple myeloid and lymphoid cell subtypes. Following a significant crossover interaction (*P* = .002; Fig. 6D), we found that this gene set was significantly down-regulated in wheel running-only mice in comparison to controls (*P* = 0.041). However, in contrast to the macrophage lactate timer transcriptome, this relatively non-resolved inflammatory state was completely reversed in wheel running mice with clodronate (*P* = 0.048). Subsequent analysis of peak inflammation genes from Kong et al. (2020) (i.e., before inflammation resolution) found no significant differences among groups (*P* values > 0.35; Fig. S3). Thus, this analysis suggests that mononuclear phagocytes might mediate a resolution gene expression program that is down-regulated by physical activity.

## Discussion

Mice that were given access to running wheels showed significantly less mammary tumor growth than control mice. These results were seen in fully immunocompetent C57BL/6 mice and in SCID mice that lack functional T cells and B cells, suggesting that innate immune cells such as mononuclear phagocytes may mediate at least part of the inhibitory effect of exercise on breast cancer growth. Consistent with that hypothesis, inhibition of mononuclear phagocytes by clodronate liposomes blocked the inhibitory effect of voluntary wheel running on mammary tumor growth. However, tumor growth was also paradoxically inhibited in mice that received clodronate liposomes in the absence of wheel-running. These results suggested that voluntary wheel running may alter a specific subset of mononuclear phagocytes in mammary tissue that would otherwise facilitate mammary tumor growth under basal conditions. Given that F4/80 (Adgre1), which is expressed by mononuclear phagocytes, was reduced by both clodronate and wheel running, we used whole-transcriptome digital cytometry to further quantify total macrophages, M1-like and M2-like macrophages, monocytes, immature DCs, and activated DCs. This analysis found higher M2-like macrophage percentages in wheel running-only mice compared to wheel runners that received clodronate and also found that monocyte percentages were significantly higher in wheel runners vs. wheel runners that received clodronate. Altogether, these data support the hypothesis that physical activity alters mononuclear phagocytes in mammary tissue when inhibiting nascent tumor growth and implicate exercise-induced changes in monocyte trafficking and macrophage differentiation as key mechanisms of such effects.

These findings suggest that voluntary physical activity augments an anti-tumor program that involves monocytes and/or M2-like macrophages. Physical activity causes non-classical monocyte influx into the bloodstream (Gabriel et al., 1994; Steppich et al., 2000; Heimbeck et al., 2010; Radom-Aizik et al., 2014), with likely subsequent extravasation into tissue after re-attachment to the endothelium (Gabriel et al., 1994). Moreover, it has recently been found that non-classical monocytes preferentially differentiate into M2-like macrophages after extravasating into sites of tissue injury (Olingy et al., 2017). Though counterintuitive, M2-like macrophages can mediate tumor cell clearance via their phagocytosis program (Gu et al., 2018). Thus, in the present study, ablation of the monocyte pool appears to have hindered an exercise-induced monocyte extravasation and subsequent differentiation into M2-like macrophages, of which either or both subsets may have exerted anti-tumor function toward nascent mammary tumor.

Exploratory analyses of the whole tissue transcriptome data suggested that mononuclear phagocytes may be part of an inflammation resolution program that is ultimately down-regulated by physical activity. Voluntary wheel running significantly reduced expression of a gene set (Kong et al., 2020; Data S4) that manifests at the resolution of a sterile inflammatory event. In association with the reversal of both monocyte and M2-like macrophage percentages in wheel runners that received clodronate, there was also a reversal of this resolution gene set in wheel runners that received clodronate. In contrast, exploratory analyses focusing on the transcriptomes for MDSC subsets and the macrophage lactate timer did not show reversals by mononuclear phagocyte ablation in wheel runners. Compared to wheel runners receiving vehicle liposomes, wheel runners receiving clodronate exhibited additively lower M-MDSC and macrophage lactate timer TRA composite scores, despite the fact that tumor signal came back up in wheel runners receiving clodronate. Thus, instead of blocking an anti-tumor pathway mediated by M-MDSCs or a singular homeostatic macrophage timer, ablation appeared to block physical activity’s inhibitory effects on a broader resolution program and subsequent nascent tumor growth. However, we emphasize that these exploratory findings need to be further confirmed in future research.

Given that Zhang et al. (2019a) found the lactate timer manifested only in M1-like (LPS-activated) macrophages, it’s possible that lactate timer composite scores in the TRA were down-regulated by wheel running because M1-like macrophages were down-regulated by wheel running. Tumors typically harbor a high accumulation of lactate, which is associated with several pro-tumor alterations, including immunosuppression (reviewed in Koelwyn et al., 2017; Zhang et al., 2019b). However, running has been found to significantly reduce lactate levels in both blood and mammary tumor in the syngeneic MC4-L2 mouse model of breast cancer, and these effects associated with inhibition of tumor growth (Aveseh, Nikooie & Aminaie, 2015). Thus, future studies are needed to further define the mechanisms by which physical activity reduces tumor lactate concentrations to possibly modulate anti-tumor immunity.

It should be noted that voluntary wheel running can be highly variable between mice (De Bono et al., 2006). Mice in the present study were given access to the wheel-hub for two weeks before tumor engraftment, to increase the likelihood that animals would provide their strongest running sessions around the time of tumor engraftment. However, given that our running wheels were not equipped to record speed and/or distance, we don’t know the exact amount of exercise by each mouse in the days leading up to engraftment or at the time surrounding engraftment. A more precise measure of running amount will allow for more precise statistical analyses in future research and further inform these results.

The finding that sedentary mice receiving clodronate liposomes exhibited a smaller but significant reduction in tumor signal may be the result of a reduction in immune tolerance, and this finding has been reported in other preclinical cancer studies with encapsulated clodronate (Van Acker et al., 2016). Outside of the malignant context, the F4/80 protein on macrophages has long been known to induce a certain level of immune tolerance to foreign antigen by mediating (in part) the activity of regulatory T cells in the periphery (Lin et al., 2005). More recently, this baseline control of immune tolerance by F4/80^+^ macrophages has been seen in allogenic graft transplantation modeling, where early macrophage ablation by clodronate liposomes (but not late ablation) results in deficient regulatory T cell generation and subsequent graft loss (Mfarrej et al., 2020). In the present study, both gene and protein expression of F4/80 were significantly reduced by clodronate. Thus, early ablation (i.e., before tumor cell engraftment) of F4/80^+^ phagocytic cells may have facilitated a reduction of immune tolerance in mammary tissue.

The novel finding of mononuclear phagocyte involvement in physical activity’s inhibitory effect extends recent concepts regarding myeloid cell involvement in anti-tumor immunotherapy for breast cancer. One of the biggest challenges facing successful immunotherapy for breast cancer is the immunologically “cold” microenvironment of the tumor, where active cytotoxic T cells are absent due to the extraordinarily immunosuppressive nature of the breast tumor (Vonderheide, Domchek & Clark, 2017). Not surprisingly, given the role of the myeloid compartment in this immunosuppression (Kim et al., 2019), checkpoint therapy with antagonism of programmed death receptor 1 (PD-1) signaling in breast cancer has been most effective when the ligand for PD-1 is expressed by tumor infiltrating immune cells (Dirix et al., 2018), of which macrophages are the most notable expressers (Loke & Allison, 2003). However, strategies to remove all tumor associated-macrophages and their monocyte precursors have been called into question (Cassetta & Kitamura, 2018) in the wake of recent findings that show a higher frequency of peripheral monocytes before onset of anti-PD-1 therapy predicts treatment responsiveness and progression-free survival (Krieg et al., 2018). Thus, we speculate that to the extent acute physical activity is able to mobilize peripheral monocytes, engagement in a certain level of structured routine exercise by cancer patients and/or survivors may serve as a highly useful adjunct that augments an otherwise lackluster success rate for anti-PD-1 immunotherapy in breast cancer. However, we emphasize that more research is needed to further define the mechanisms of physical activity’s effects on mononuclear phagocytes in the malignant context before moving toward such anti-tumor immunotherapy studies in cancer patients and/or survivors.

## Conclusions

To conclude, these data support the hypothesis that physical activity inhibits nascent tumor growth by altering mononuclear phagocyte biology in mammary tissue and implicate exercise-induced changes in monocyte trafficking and macrophage differentiation as a key mechanism of such effects. Future experiments will be needed to further pinpoint the mechanisms by which these effects are produced.

##  Supplemental Information

10.7717/peerj.10725/supp-1 Figure S1 Schematic representation of FUG2ALucW FUG2ALucW has the CMV enhancer for the U3 region of the 5′ LTR. ΔU3 denotes a deletion in the U3 region of the 3′ LTR that renders the 5′ LTR of the integrated provirus transcriptionally inactive. FUG2ALucW has the Ubiqutin-C promoter as an internal promoter to express the fusion protein (G2ALuc) of EGFP, self-cleaving T2A sequence derived from Thosea asigna virus, and firefly luciferase (Ibrahimi et al., 2009). FUG2ALucW has the central polypurine tract (cPPT) and the Woodchuck hepatitis virus post transcriptional element (WRE).

10.7717/peerj.10725/supp-2 Figure S2 Neutrophil proportions in mammary tissue as estimated by CIBERSORTx Neutrophil proportions in mammary tissue as estimated by CIBERSORTx with the ImmuCC reference gene set at Day 1 in the immunocompetent C57BL/6-EO771 model of breast cancer. Mean ± SEM and individual data points, with *n* = 5–6 mice per group. Clodro: Clodronate.

10.7717/peerj.10725/supp-3 Figure S3 TRA composite scores in the immunocompetent C57BL/6-EO771 model of breast cancer as a function of voluntary wheel running × clodronate treatment at Day 1 for peak inflammation from Kong et al. (2020) Peak inflammation from Kong et al. (2020) were genes up-regulated by 50% at peak inflammation vs. baseline, before subsequent resolution of inflammation (Supplementary Data 6). Mean ± SEM and individual data points, with *n* = 5–6 mice per group. Clodro: Clodronate.

10.7717/peerj.10725/supp-4 Data S1 Raw data

10.7717/peerj.10725/supp-5 Data S2 ImmuCC Gene Signature Matrix from Chen et al. (2017)

10.7717/peerj.10725/supp-6 Data S3 Myeloid-Derived Suppressor Cell Reference Genes from Alshetaiwi et al. (2020)

10.7717/peerj.10725/supp-7 Data S4 Inflammation Resolution Reference Genes from Kong et al. (2020)

10.7717/peerj.10725/supp-8 Data S5 Macrophage Lactate Timer Genes from Zhang et al. (2019)

## Additional Information and Declarations

### DNA Deposition

The following information was supplied regarding the deposition of DNA sequences: RNAseq data are accessible in the US National Center for Biotechnology Information Gene Expression Omnibus (GEO): GSE150620.

### Data Availability

The following information was supplied regarding data availability: Raw data is available in the Supplemental Files.

## References

[ref-1] Abdalla DR, Aleixo AA, Murta EF, Michelin MA (2014). Innate immune response adaptation in mice subjected to administration of DMBA and physical activity. Oncology Letters.

[ref-2] Adlam D, De Bono JP, Danson EJ, Zhang MH, Casadei B, Paterson DJ, Channon KM (2011). Telemetric analysis of haemodynamic regulation during voluntary exercise training in mouse models. Experimental Physiology.

[ref-3] Alshetaiwi H, Pervolarakis N, McIntyre LL, Ma D, Nguyen Q, Rath JA, Nee K, Hernandez G, Evans K, Torosian L, Silva A, Walsh C, Kessenbrock K (2020). Defining the emergence of myeloid-derived suppressor cells in breast cancer using single-cell transcriptomics. Science Immunology.

[ref-4] Aveseh M, Nikooie R, Aminaie M (2015). Exercise-induced changes in tumour LDH-B and MCT1 expression are modulated by oestrogen-related receptor alpha in breast cancer-bearing BALB/c mice. Journal de Physiologie.

[ref-5] Ballard-Barbash R, Friedenreich CM, Courneya KS, Siddiqi SM, McTiernan A, Alfano CM (2012). Physical activity, biomarkers, and disease outcomes in cancer survivors: a systematic review. Journal of the National Cancer Institute.

[ref-6] Bianco TM, Abdalla DR, Desidério CS, Thys S, Simoens C, Bogers JP, Murta EFC, Michelin MA (2017). The influence of physical activity in the anti-tumor immune response in experimental breast tumor. Immunology Letters.

[ref-7] Cao H, Wolff RG, Meltzer MS, Crawford RM (1989). Differential regulation of class II MHC determinants on macrophages by IFN-gamma and IL-4. Journal of Immunology.

[ref-8] Cassetta L, Kitamura T (2018). Targeting tumor-associated macrophages as a potential strategy to enhance the response to immune checkpoint inhibitors. Frontiers in Cell and Developmental Biology.

[ref-9] Chen Z, Huang A, Sun J, Jiang T, Qin FX, Wu A (2017). Inference of immune cell composition on the expression profiles of mouse tissue. Scientific Reports.

[ref-10] Chua BA, Ngo JA, Situ K, Morizono K (2019). Roles of phosphatidylserine exposed on the viral envelope and cell membrane in HIV-1 replication. Cell Communication and Signaling.

[ref-11] Da Silva AR, Malafaia G, Menezes IPP (2017). biotools: an R function to predict spatial gene diversity via an individual-based approach. Genetics and Molecular Research.

[ref-12] De Bono JP, Adlam D, Paterson DJ, Channon KM (2006). Novel quantitative phenotypes of exercise training in mouse models. American Journal of Physiology.

[ref-13] Dirix LY, Takacs I, Jerusalem G, Nikolinakos P, Arkenau HT, Forero-Torres A, Boccia R, Lippman ME, Somer R, Smakal M, Emens LA, Hrinczenko B, Edenfield W, Gurtler J, Von Heydebreck A, Grote HJ, Chin K, Hamilton EP (2018). Avelumab, an anti-PD-L1 antibody, in patients with locally advanced or metastatic breast cancer: a phase 1b JAVELIN Solid Tumor study. Breast Cancer Research and Treatment.

[ref-14] Friedenreich CM, Neilson HK, Farris MS, Courneya KS (2016). Physical activity and cancer outcomes: a precision medicine approach. Clinical Cancer Research.

[ref-15] Friendly M, Fox J (2017). https://CRAN.R-project.org/package=candisc.

[ref-16] Gabriel H, Urhausen A, Brechtel L, Müller HJ, Kindermann W (1994). Alterations of regular and mature monocytes are distinct, and dependent of intensity and duration of exercise. European Journal of Applied Physiology and Occupational Physiology.

[ref-17] Gibert-Ramos A, López C, Bosch R, Fontoura L, Bueno G, García-Rojo M, Berenguer M, Lejeune M (2019). Immune response profile of primary tumour, sentinel and non-sentinel axillary lymph nodes related to metastasis in breast cancer: an immunohistochemical point of view. Histochemistry and Cell Biology.

[ref-18] Gordon S, Hamann J, Lin HH, Stacey M (2011). F4/80 and the related adhesion-GPCRs. European Journal of Immunology.

[ref-19] Gu S, Ni T, Wang J, Liu Y, Fan Q, Wang Y, Huang T, Chu Y, Sun X, Wang Y (2018). CD47 blockade inhibits tumor progression through promoting phagocytosis of tumor cells by M2 polarized macrophages in endometrial cancer. Journal of Immunology Research.

[ref-20] Guilliams M, Ginhoux F, Jakubzick C, Naik SH, Onai N, Schraml BU, Segura E, Tussiwand R, Yona S (2014). Dendritic cells, monocytes and macrophages: a unified nomenclature based on ontogeny. Nature Reviews Immunology.

[ref-21] Hartig SM (2013). Basic image analysis and manipulation in ImageJ. Current Protocols in Molecular Biology.

[ref-22] Heimbeck I, Hofer TP, Eder C, Wright AK, Frankenberger M, Marei A, Boghdadi G, Scherberich J, Ziegler-Heitbrock L (2010). Standardized single-platform assay for human monocyte subpopulations: lower CD14+CD16++ monocytes in females. Cytometry A.

[ref-23] Hojman P, Gehl J, Christensen JF, Pedersen BK (2018). Molecular mechanisms linking exercise to cancer prevention and treatment. Cell Metabolism.

[ref-24] Ibrahimi A, Vande Velde G, Reumers V, Toelen J, Thiry I, Vandeputte C, Vets S, Deroose C, Bormans G, Baekelandt V, Debyser Z, Gijsbers R (2009). Highly efficient multicistronic lentiviral vectors with peptide 2A sequences. Human Gene Therapy.

[ref-25] Kawanishi N, Yano H, Yokogawa Y, Suzuki K (2010). Exercise training inhibits inflammation in adipose tissue via both suppression of macrophage infiltration and acceleration of phenotypic switching from M1 to M2 macrophages in high-fat-diet-induced obese mice. Exercise Immunology Review.

[ref-26] Kim IS, Gao Y, Welte T, Wang H, Liu J, Janghorban M, Sheng K, Niu Y, Goldstein A, Zhao N, Bado I, Lo HC, Toneff MJ, Nguyen T, Bu W, Jiang W, Arnold J, Gu F, He J, Jebakumar D, Walker K, Li Y, Mo Q, Westbrook TF, Zong C, Rao A, Sreekumar A, Rosen JM, Zhang XH (2019). Immuno-subtyping of breast cancer reveals distinct myeloid cell profiles and immunotherapy resistance mechanisms. Nature Cell Biology.

[ref-27] Kizaki T, Takemasa T, Sakurai T, Izawa T, Hanawa T, Kamiya S, Haga S, Imaizumi K, Ohno H (2008). Adaptation of macrophages to exercise training improves innate immunity. Biochemical and Biophysical Research Communications.

[ref-28] Koelwyn GJ, Quail DF, Zhang X, White RM, Jones LW (2017). Exercise-dependent regulation of the tumour microenvironment. Nature Reviews Cancer.

[ref-29] Kong JS, Park JH, Yoo SA, Kim KM, Bae YJ, Park YJ, Cho CS, Hwang D, Kim WU (2020). Dynamic transcriptome analysis unveils key proresolving factors of chronic inflammatory arthritis. Journal of Clinical Investigation.

[ref-30] Krieg C, Nowicka M, Guglietta S, Schindler S, Hartmann FJ, Weber LM, Dummer R, Robinson MD, Levesque MP, Becher B (2018). High-dimensional single-cell analysis predicts response to anti-PD-1 immunotherapy. Nature Medicine.

[ref-31] Lamkin DM, Srivastava S, Bradshaw KP, Betz JE, Muy KB, Wiese AM, Yee SK, Waggoner RM, Arevalo JMG, Yoon AJ, Faull KF, Sloan EK, Cole SW (2019). C/EBP *β* regulates the M2 transcriptome in *β*-adrenergic-stimulated macrophages. Brain, Behavior, and Immunity.

[ref-32] Lamkin DM, Sung HY, Yang GS, David JM, Ma JC, Cole SW, Sloan EK (2015). *α*2-Adrenergic blockade mimics the enhancing effect of chronic stress on breast cancer progression. Psychoneuroendocrinology.

[ref-33] Lin HH, Faunce DE, Stacey M, Terajewicz A, Nakamura T, Zhang-Hoover J, Kerley M, Mucenski ML, Gordon S, Stein-Streilein J (2005). The macrophage F4/80 receptor is required for the induction of antigen-specific efferent regulatory T cells in peripheral tolerance. Journal of Experimetnal Medicine.

[ref-34] Lois C, Hong EJ, Pease S, Brown EJ, Baltimore D (2002). Germline transmission and tissue-specific expression of transgenes delivered by lentiviral vectors. Science.

[ref-35] Loke P, Allison JP (2003). PD-L1 and PD-L2 are differentially regulated by Th1 and Th2 cells. Proceedings of the National Academy of Sciences of the United States of America.

[ref-36] Ma H, Xu X, Clague J, Lu Y, Togawa K, Wang SS, Clarke CA, Lee E, Park HL, Sullivan-Halley J, Neuhausen SL, Bernstein L (2016). Recreational physical activity and risk of triple negative breast cancer in the California Teachers Study. Breast Cancer Research.

[ref-37] Mantovani A, Marchesi F, Malesci A, Laghi L, Allavena P (2017). Tumour-associated macrophages as treatment targets in oncology. Nature Reviews Clinical Oncology.

[ref-38] McClellan JL, Steiner JL, Day SD, Enos RT, Davis MJ, Singh UP, Murphy EA (2014). Exercise effects on polyp burden and immune markers in the ApcMin/+ mouse model of intestinal tumorigenesis. International Journal of Oncology.

[ref-39] McTiernan A (2008). Mechanisms linking physical activity with cancer. Nature Reviews Cancer.

[ref-40] Meyer D, Dimitriadou E, Hornik K, Weingessel A, Leisch F (2019). https://CRAN.R-project.org/package=e1071.

[ref-41] Mfarrej B, Jofra T, Morsiani C, Gagliani N, Fousteri G, Battaglia M (2020). Key role of macrophages in tolerance induction via T regulatory type 1 (Tr1) cells. Clinical and Experimental Immunology.

[ref-42] Moore SC, Lee IM, Weiderpass E, Campbell PT, Sampson JN, Kitahara CM, Keadle SK, Arem H, Berrington de Gonzalez A, Hartge P, Adami HO, Blair CK, Borch KB, Boyd E, Check DP, Fournier A, Freedman ND, Gunter M, Johannson M, Khaw KT, Linet MS, Orsini N, Park Y, Riboli E, Robien K, Schairer C, Sesso H, Spriggs M, Van Dusen R, Wolk A, Matthews CE, Patel AV (2016). Association of leisure-time physical activity with risk of 26 types of cancer in 1.44 million adults. JAMA Internal Medicine.

[ref-43] Murphy EA, Davis JM, Brown AS, Carmichael MD, Mayer EP, Ghaffar A (2004). Effects of moderate exercise and oat beta-glucan on lung tumor metastases and macrophage antitumor cytotoxicity. Journal of Applied Physiology.

[ref-44] Murray PJ (2020). On macrophage diversity and inflammatory metabolic timers. Nature Reviews Immunology.

[ref-45] Newman AM, Liu CL, Green MR, Gentles AJ, Feng W, Xu Y, Hoang CD, Diehn M, Alizadeh AA (2015). Robust enumeration of cell subsets from tissue expression profiles. Nature Methods.

[ref-46] Newman AM, Steen CB, Liu CL, Gentles AJ, Chaudhuri AA, Scherer F, Khodadoust MS, Esfahani MS, Luca BA, Steiner D, Diehn M, Alizadeh AA (2019). Determining cell type abundance and expression from bulk tissues with digital cytometry. Nature Biotechnology.

[ref-47] Olingy CE, Dinh HQ, Hedrick CC (2019). Monocyte heterogeneity and functions in cancer. Journal of Leukocyte Biology.

[ref-48] Olingy CE, San Emeterio CL, Ogle ME, Krieger JR, Bruce AC, Pfau DD, Jordan BT, Peirce SM, Botchwey EA (2017). Non-classical monocytes are biased progenitors of wound healing macrophages during soft tissue injury. Scientific Reports.

[ref-49] Ortega-Gómez A, Perretti M, Soehnlein O (2013). Resolution of inflammation: an integrated view. EMBO Molecular Medicine.

[ref-50] Peake JM (2002). Exercise-induced alterations in neutrophil degranulation and respiratory burst activity: possible mechanisms of action. Exercise Immunology Review.

[ref-51] Pedersen L, Idorn M, Olofsson GH, Lauenborg B, Nookaew I, Hansen RH, Johannesen HH, Becker JC, Pedersen KS, Dethlefsen C, Nielsen J, Gehl J, Pedersen BK, Straten PThor, Hojman P (2016). Voluntary running suppresses tumor growth through epinephrine- and il-6-dependent NK cell mobilization and redistribution. Cell Metabolism.

[ref-52] Powell ND, Sloan EK, Bailey MT, Arevalo JM, Miller GE, Chen E, Kobor MS, Reader BF, Sheridan JF, Cole SW (2013). Social stress up-regulates inflammatory gene expression in the leukocyte transcriptome via *β*-adrenergic induction of myelopoiesis. Proceedings of the National Academy of Sciences of the United States of America.

[ref-53] Radom-Aizik S, Zaldivar Jr FP, Haddad F, Cooper DM (2014). Impact of brief exercise on circulating monocyte gene and microRNA expression: implications for atherosclerotic vascular disease. Brain, Behavior, and Immunity.

[ref-54] R Core Team (2019). R: a language and environment for statistical computing.

[ref-55] Schneider CA, Rasband WS, Eliceiri KW (2012). NIH Image to ImageJ: 25 years of image analysis. Nature Methods.

[ref-56] Shantsila E, Tapp LD, Wrigley BJ, Montoro-Garcia S, Ghattas A, Jaipersad A, Lip GY (2012). The effects of exercise and diurnal variation on monocyte subsets and monocyte-platelet aggregates. European Journal of Clinical Investigation.

[ref-57] Siegel RL, Miller KD, Jemal A (2020). Cancer statistics, 2020. CA: A Cancer Journal for Clinicians.

[ref-58] Singmann H, Bolker B, Westfall J, Aust F, Ben-Shachar MS (2019). https://CRAN.R-project.org/package=afex.

[ref-59] Sinha P, Clements VK, Bunt SK, Albelda SM, Ostrand-Rosenberg S (2007). Cross-talk between myeloid-derived suppressor cells and macrophages subverts tumor immunity toward a type 2 response. Journal of Immunology.

[ref-60] Steppich B, Dayyani F, Gruber R, Lorenz R, Mack M, Ziegler-Heitbrock HW (2000). Selective mobilization of CD14(+)CD16(+) monocytes by exercise. American Journal of Physiology. Cell Physiology.

[ref-61] Sugiura H, Nishida H, Sugiura H, Mirbod SM (2002). Immunomodulatory action of chronic exercise on macrophage and lymphocyte cytokine production in mice. Acta Physiologica Scandinavica.

[ref-62] Ullman-Cullere MH, Foltz CJ (1999). Body condition scoring: a rapid and accurate method for assessing health status in mice. Laboratory Animal Science.

[ref-63] Vainio H, Bianchini F (2002). IARC handbooks of cancer prevention, weight control, and physical activity.

[ref-64] Van Acker HH, Anguille S, Willemen Y, Smits EL, Van Tendeloo VF (2016). Bisphosphonates for cancer treatment: mechanisms of action and lessons from clinical trials. Pharmacology and Therapeutics.

[ref-65] Vonderheide RH, Domchek SM, Clark AS (2017). Immunotherapy for breast cancer: what are we missing?. Clinical Cancer Research.

[ref-66] Walsh NP, Gleeson M, Shephard RJ, Gleeson M, Woods JA, Bishop NC, Fleshner M, Green C, Pedersen BK, Hoffman-Goetz L, Rogers CJ, Northoff H, Abbasi A, Simon P (2011). Position statement. Part one: immune function and exercise. Exercise Immunology Review.

[ref-67] Weisser SB, Rooijen Nvan, Sly LM (2012). Depletion and reconstitution of macrophages in mice. Journal of Visualized Experiments.

[ref-68] Wennerberg E, Lhuillier C, Rybstein MD, Dannenberg K, Rudqvist NP, Koelwyn GJ, Jones LW, Demaria S (2020). Exercise reduces immune suppression and breast cancer progression in a preclinical model. Oncotarget.

[ref-69] Zhang X, Ashcraft KA, Warner ABetof, Nair SK, Dewhirst MW (2019b). Can exercise-induced modulation of the tumor physiologic microenvironment improve antitumor immunity?. Cancer Research.

[ref-70] Zhang D, Tang Z, Huang H, Zhou G, Cui C, Weng Y, Liu W, Kim S, Lee S, Perez-Neut M, Ding J, Czyz D, Hu R, Ye Z, He M, Zheng YG, Shuman HA, Dai L, Ren B, Roeder RG, Becker L, Zhao Y (2019a). Metabolic regulation of gene expression by histone lactylation. Nature.

[ref-71] Zielinski MR, Muenchow M, Wallig MA, Horn PL, Woods JA (2004). Exercise delays allogeneic tumor growth and reduces intratumoral inflammation and vascularization. Journal of Applied Physiology.

