# Supplementary material for: Physical activity modulates mononuclear phagocytes in mammary tissue and inhibits tumor growth in mice
Source: PeerJ. 2021 Jan 19;9:e10725. doi: 10.7717/peerj.10725 (PMC7821756; doi:10.7717/peerj.10725)
Supplement: Figure S1 — FUG2ALucW has the CMV enhancer for the U3 region of the 5′ LTR. ΔU3 denotes a deletion in the U3 region of the 3′ LTR that renders the 5′ LTR of the integrated provirus transcriptionally inactive. FUG2ALucW has the Ubiqutin-C promoter as an internal promoter to express the fusion protein (G2ALuc) of EGFP, self-cleaving T2A sequence derived from Thosea asigna virus, and firefly luciferase (Ibrahimi et al., 2009). FUG2ALucW has the central polypurine tract (cPPT) and the Woodchuck hepatitis virus post transcriptional element (WRE). [file peerj-09-10725-s001.pdf]

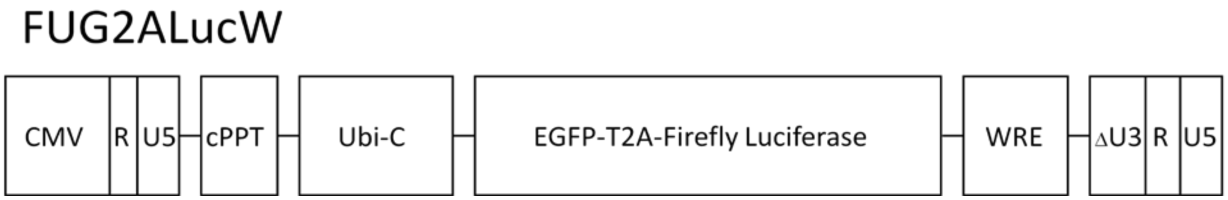

Supplementary Figure 1. Schematic representation of FUG2ALucW. FUG2ALucW has the CMV enhancer for the U3 region of the 5' LTR. ΔU3 denotes a deletion in the U3 region of the 3' LTR that renders the 5' LTR of the integrated provirus transcriptionally inactive. FUG2ALucW has the Ubiqutin-C promoter as an internal promoter to express the fusion protein (G2ALuc) of EGFP, self-cleaving T2A sequence derived from Thosea asigna virus, and firefly luciferase (Ibrahimi et al., 2009). FUG2ALucW has the central polypurine tract (cPPT) and the Woodchuck hepatitis virus post transcriptional element (WRE).
