# Supplementary material for: Physical activity modulates mononuclear phagocytes in mammary tissue and inhibits tumor growth in mice
Source: PeerJ. 2021 Jan 19;9:e10725. doi: 10.7717/peerj.10725 (PMC7821756; doi:10.7717/peerj.10725)
Supplement: Figure S2 — Neutrophil proportions in mammary tissue as estimated by CIBERSORTx with the ImmuCC reference gene set at Day 1 in the immunocompetent C57BL/6-EO771 model of breast cancer. Mean ± SEM and individual data points, with n = 5–6 mice per group. Clodro: Clodronate. [file peerj-09-10725-s002.pdf]

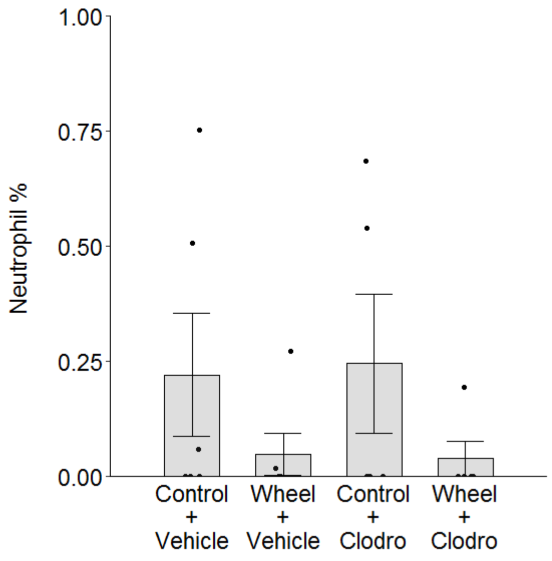

Supplementary Figure 2. Neutrophil proportions in mammary tissue as estimated by CIBERSORTx with the ImmuCC reference gene set at Day 1 in the immunocompetent C57BL/6-EO771 model of breast cancer, with n = 5-6 mice per group. Clodro: Clodronate.
