# Supplementary material for: Physical activity modulates mononuclear phagocytes in mammary tissue and inhibits tumor growth in mice
Source: PeerJ. 2021 Jan 19;9:e10725. doi: 10.7717/peerj.10725 (PMC7821756; doi:10.7717/peerj.10725)
Supplement: Figure S3 — Peak inflammation from Kong et al. (2020) were genes up-regulated by 50% at peak inflammation vs. baseline, before subsequent resolution of inflammation (Supplementary Data 6). Mean ± SEM and individual data points, with n = 5–6 mice per group. Clodro: Clodronate. [file peerj-09-10725-s003.pdf]

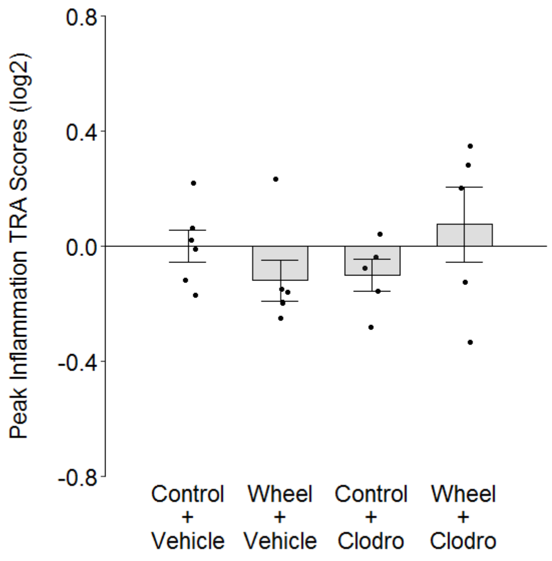

Supplementary Figure 3. TRA composite scores in the immunocompetent C57BL/6-EO771 model of breast cancer as a function of voluntary wheel running x clodronate treatment at Day 1 for peak inflammation from Kong et al. (2020) (genes up-regulated by 50% at peak inflammation vs. baseline, before subsequent resolution of inflammation) (Supplementary Data 6). Mean ± SEM and individual data points, with n = 5-6 mice per group. Clodro: Clodronate.
